# Supplementary material for: Machine Learning Model Based on Optimized Radiomics Feature from 18F-FDG-PET/CT and Clinical Characteristics Predicts Prognosis of Multiple Myeloma: A Preliminary Study
Source: J Clin Med. 2023 Mar 15;12(6):2280. doi: 10.3390/jcm12062280 (PMC10059677; doi:10.3390/jcm12062280)
Supplement: Supplementary file 1 [file jcm-12-02280-s001.zip › jcm-2136939-supplementary.pdf]

SUPPLEMENTAL MATERIALS

Table S1: IBSI reporting structure of the study as of the Imaging Biomarker Standardization Initiative (IBSI) guidelines (1)

**Patient**	
Volume of Interest	The spine and pelvis
Patient Preparation	With newly diagnosed multiple myeloma who underwent 18F-FDG-PET/CT between 2014 and 2019 in Renji hospital.
Radiotracer	[^{18}F]FDG
**Acquisition and Reconstruction**	
Protocol	With newly diagnosed multiple myeloma who underwent 18F-FDG-PET/CT between 2014 and 2019 in Renji hospital.
Scanner type	Siemens Biograph-64 mCT scanner
[^{18}F]FDG PET	- Static<br>- Scanning used 5 to 6 beds<br>- Each bed acquisition 2 minutes<br>- Using 3 iterations and 24 subsets of ordered subset expectation maximization algorithm(OSEM)<br>- By PSF, FWHM 2mm<br>- 2.75 x 3.12 x 3.12 mm voxel size
CT	- 120kV<br>- 80~120mA<br>- 2 x 0.977 x 0.977 mm voxel size
**Image Co-registration**	
Software	3D Slicer 4.11(General Registration(Elastix)) (https://www.slicer.org)
Co-registration step 1	Automated as of DICOM coordinate parameters
Co-registration step 2	Optional in case of misalignments detected: CT imaging to [^{18}F]FDG PET via automated rigid registration + manual correction if necessary
**Segmentation**	
Software	3D Slicer 4.11(Segment Editor)
VOI definition	Standard semi-automated iso-count 3D based on side-by-side viewing PET/CT and respective annotated histopathological slices of the spine and pelvis.
Number of experts	2+1 (2 nuclear medicine experts participated in independent delineations, followed by 1 senior nuclear medicine specialist cross-validation and if necessary, modification of first-round results)
Reference image	CT
**Image / VOI interpolation**	

Method	Kriging interpolation in 3D (2), including nearest neighbors within distance of voxel size main diagonal + epsilon.
Grid	Align by center
Extrapolation beyond original image	Neighbor distance search calculated as original voxel size main diagonal + epsilon. Missing value: image minimum
Voxel dimensions	1.0 x 1.0 x 1.0 mm
Partially masked voxels (VOI)	Taken if more than half of original voxel area included
**Discretization**	
Method	Fixed bin width, variable number of bins
Bin width	- $[^{18}F]$FDG: 0.25
**Image biomarker computation / Parameters**	
Biomarker set	- Intensity features (21 per image): Minimum, Maximum, Mean, Sum, Variance, Skewness, Kurtosis, Median, 10th intensity percentile, 90th intensity percentile, Interquartile range, Range, Intensity-based mean absolute deviation, Intensity-based robust mean absolute deviation, Intensity-based median absolute deviation, Intensity-based coefficient of variation, Intensity-based quartile coefficient of dispersion, Energy, Root mean square intensity, Local intensity peak, Global intensity peak<br>- Histogram features (19 per image): Mean discretised intensity, Discretised intensity variance, Discretised intensity skewness, (Excess) discretised intensity kurtosis, Median discretised intensity, Minimum discretised intensity, Maximum discretised intensity, Intensity histogram mode, Intensity histogram mean absolute deviation, Intensity histogram robust mean absolute deviation, Intensity histogram median absolute deviation, Intensity histogram coefficient of variation, Intensity histogram quartile coefficient of dispersion, Discretised intensity entropy, Discretised intensity uniformity, Maximum histogram gradient, Maximum histogram gradient intensity, Minimum histogram gradient, Minimum histogram gradient intensity<br>- Intensity histogram features (6 per image): Volume at intensity fraction 10%, Volume at intensity fraction 90%, Intensity at volume fraction 10%, Intensity at volume fraction 90%, Volume fraction difference between intensity fractions, Intensity fraction difference between volume fractions<br>- GLCM features (24 per image): Joint maximum, Joint average, Joint variance, Joint entropy, Difference Average, Difference variance, Difference entropy, Sum average, Sum variance, Sum entropy, Angular second moment, Contrast, Dissimilarity, Inverse difference, Normalised inverse difference, Inverse difference moment, Normalised inverse difference moment, Inverse variance, Correlation, Autocorrelation, Cluster shade, Cluster prominence, Information correlation 1, Information correlation 2

	- Morphological features (13 per image): Volume (mesh), Surface area (mesh), Surface to volume ratio, Compactness 1, Compactness 2, Spherical disproportion, Sphericity, Asphericity, Maximum 3D diameter, Volume density (axis-aligned bounding box), Area density (axis-aligned bounding box), Volume density (convex hull), Area density (convex hull)<br>- GLSZM features (16 per image): Small zone emphasis, Large zone emphasis, Low grey level zone emphasis, High grey level zone emphasis, Small zone low grey level emphasis, Small zone high grey level emphasis, Large zone low grey level emphasis, Large zone high grey level emphasis, Grey level non-uniformity, Normalised grey level non-uniformity, Zone size non-uniformity, Normalised zone size non-uniformity, Zone percentage, Grey level variance, Zone size variance, Zone size entropy<br>- NGTDM features (5 per image): Coarseness, Contrast, Busyness, Complexity, Strength<br>- GLRLM features (16 per image): Short runs emphasis, Long runs emphasis, Low grey level run emphasis, High grey level run emphasis, Short run low grey level emphasis, Short run high grey level emphasis, Long run low grey level emphasis, Long run high grey level emphasis, Grey level non-uniformity, Normalised grey level non-uniformity, Run length non-uniformity, Normalised run length non-uniformity, Run percentage, Grey level variance, Run length variance, Run entropy<br>- NGLDM features (17 per image): Low dependence emphasis, High dependence emphasis, Low grey level count emphasis, High grey level count emphasis, Low dependence low grey level emphasis, Low dependence high grey level emphasis, High dependence low grey level emphasis, High dependence high grey level emphasis, Grey level non-uniformity, Normalised grey level non-uniformity, Dependence count non-uniformity, Normalised dependence count non-uniformity, Dependence count percentage, Grey level variance, Dependence count variance, Dependence count entropy, Dependence count energy<br>- GLDZM features (16 per image): Small distance emphasis, Large distance emphasis, Low grey level zone emphasis, High grey level zone emphasis, Small distance low grey level emphasis, Small distance high grey level emphasis, Large distance low grey level emphasis, Large distance high grey level emphasis, Grey level non-uniformity, Normalised grey level non-uniformity, Zone distance non-uniformity, Normalised zone distance non-uniformity, Zone percentage, Grey level variance, Zone distance variance, Zone distance entropy
Software	pyradiomics ver. 3.0.1(https://github.com/Radiomics/pyradiomics)(3), developed and validated based on IBSI guidelines and reference datasets (1)..

References

1. Zwanenburg A, Leger S, Vallières M, Löck S, Initiative for the IBS. Image biomarker standardisation initiative. arXiv [Internet]. 2016;(November). Available from: http://arxiv.org/abs/1612.07003

2. Stytz MR, Parrott RW. Using kriging for 3d medical imaging. Comput Med Imaging Graph. 1993;17(6):421–42.

3. van Griethuysen, J. J. M., Fedorov, A., Parmar, C., Hosny, A., Aucoin, N., Narayan, V., Beets-Tan, R. G. H., Fillon-Robin, J. C., Pieper, S., Aerts, H. J. W. L. (2017). Computational Radiomics System to Decode the Radiographic Phenotype. Cancer Research, 77(21), e104–e107. `https://doi.org/10.1158/0008-5472.
